# Supplementary material for: A neural network model for timing control with reinforcement
Source: Front Comput Neurosci. 2022 Oct 5;16:918031. doi: 10.3389/fncom.2022.918031 (PMC9579423; doi:10.3389/fncom.2022.918031)
Supplement: Supplementary file 1 [file Data_Sheet_1.docx]

***Supplementary Materials***

1. **Supplementary Data and Figures**

**Optimal control of the synaptic variance.**

Let’s assume that the coefficients have constant means in the vAR, and the system was able to inject noise of various amplitudes to maximize the gain in the next trial. Under those assumptions, the expected error and variance of the error can be expressed as

$$\hat{e}_{n}=\sum_{p} \hat{\omega_{i}}e_{n-i}$$

$$\Sigma^{2}=\text{cov}\left( e_{n} \right)=\sum_{i}^{p} \text{cov}\left( \omega_{i} \right)e_{n-i}^{2}+n_{0}^{2}$$

Hence, the probability was computed by integrating the error distribution and feedback function, shown in Supplementary Figure 1A.

$$E\left( r \right)=\frac{1}{\sqrt{2\pi}\Sigma}\int_{-\infty}^{+\infty} r\left( x \right)exp\left( -\frac{\left( x-\hat{e} \right)^{2}}{2\Sigma^{2}} \right)dx$$

$$=\frac{1}{\sqrt{2\pi}\Sigma}\int_{-D}^{+D} r\left( x \right)exp\left( -\frac{\left( x-\hat{e} \right)^{2}}{2\Sigma^{2}} \right)dx$$

$$=\frac{1}{2}\left[ erf\left( \frac{D+\hat{e}}{\sqrt{2}\Sigma} \right)+erf\left( \frac{D-\hat{e}}{\sqrt{2}\Sigma} \right) \right]$$

In which $r\left( x \right)$ is the square feedback function and D is the acceptance window width. erf(.) is the integral of the Gaussian. To solve the optimization, we have

$$\partial E\left( r \right)/\partial\Sigma=-\frac{1}{\sqrt{2\pi}}\left( \frac{D+\hat{e}}{\Sigma^{2}}\text{exp}\left[ -\frac{\left( D+\hat{e} \right)^{2}}{2\Sigma^{2}} \right]+\frac{D-\hat{e}}{\Sigma^{2}}\text{exp}\left[ -\frac{\left( D-\hat{e} \right)^{2}}{2\Sigma^{2}} \right] \right)$$

When $D>\left| \hat{e} \right|$, $E\left( r \right)$ is a monotonically increasing function as $\Sigma$ decreasing. However, $\Sigma$ is bounded and the optimal $\Sigma$ is therefore chosen to be minimal. When $D\leq\left| \hat{e} \right|$, we have

$$\partial E\left( r \right)/\partial\Sigma=0\Rightarrow\Sigma^{2}=2D\left| \hat{e} \right|/ln\frac{\left| D+\hat{e} \right|}{\left| D-\hat{e} \right|}$$

We have the optimal variance ($\Sigma_{\text{Analytical}}^{2}$) and the model choice of variance ($\Sigma_{\text{Approx}}^{2}$) as the function of expected error ($\hat{e}$), which is shown in Supplementary Figure 1B. In this illustration, without losing generality, we will assume the first term dominates in the AR relationship, i.e. $\omega_{1}\gg\omega_{2},\ldots$.

$$\Sigma_{Analytical}^{2}\left( \hat{e} \right)=\left\{ \begin{aligned} \sigma_{0}^{2} \\ 2D\left| \hat{e} \right|/ln\left| \frac{D+\hat{e}}{D-\hat{e}} \right| \end{aligned} {\left| \hat{e} \right|\leq D \atop\left| \hat{e} \right|>D} \right.$$

$$\Sigma_{Approx}^{2}\left( \hat{e} \right)=\left\{ \begin{aligned} \frac{\hat{e}^{2}\sigma_{+}^{2}}{\omega_{1}^{2}}+\sigma_{0}^{2} \\ \frac{\hat{e}^{2}\sigma_{-}^{2}}{\omega_{1}^{2}}+\sigma_{0}^{2} \end{aligned} {\left| \hat{e} \right|\leq D \atop\left| \hat{e} \right|>D} \right.$$

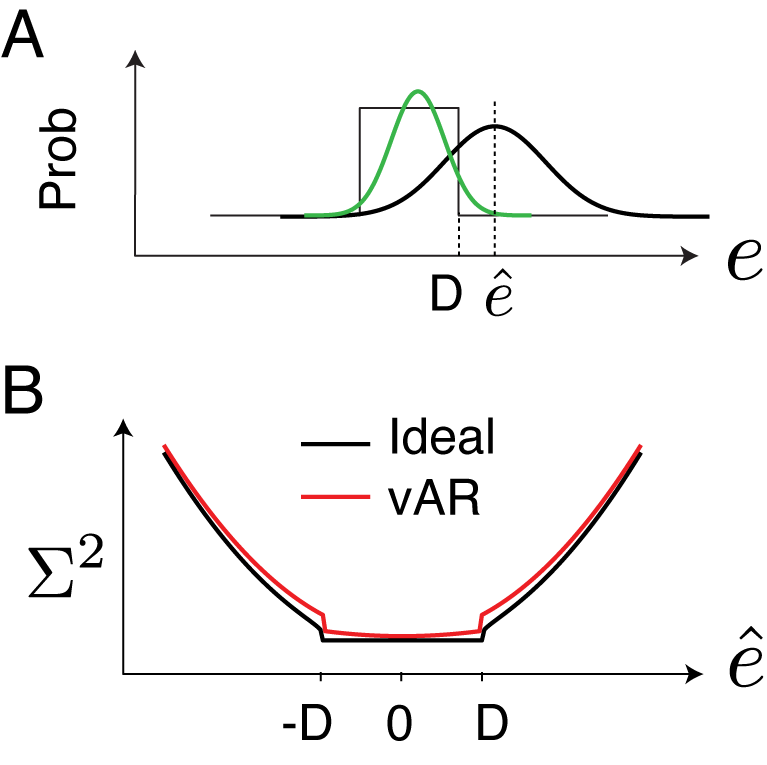


**Supplementary Figure 1**. Optimal variance adjustment. (A) The expected rate of receiving positive feedback is obtained by combining the feedback (Square shape) and the error distribution (Gaussian). In an ideal scenario, the variance of the error could be adjusted freely. It decreases when the estimated error size is small (green) and increases when the error size is large (black). (B) The relationship between variance size and mean of error. For the ideal case, the variance was computed analytically according to the equation. In our model, we have adopted a simple binary choice of variance, and it resulted in a close approximation to the ideal case.


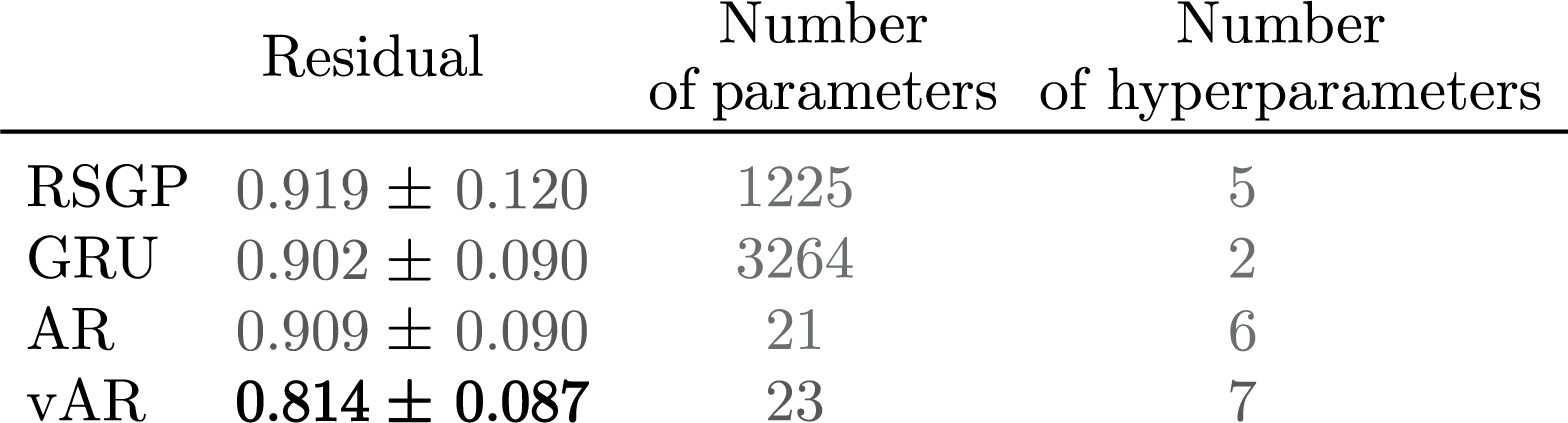


**Supplementary Table 1**. Summary of all models on behavioral data. In the RSGP, the hyperparameters are $\{l_{se},\sigma_{se},l_{rs},\sigma_{rs},\sigma_{0}\}$. In the GRU model including reward as its input, the hyperparameters are dimensional of the hidden unit and regularization factor $\{ h, \lambda\}$. In the vAR, the hyperparameters are the dimension of the memory unit and the ratio of the variance, plus the four regularization coefficients $\{p,\sigma_{+/-},\sigma_{0},C_{1},C_{2},\lambda_{1},\lambda_{2}\}$. In AR, they are the dimension of memory units and additive noise at the output $\{p,\sigma_{0},C_{1},C_{2},\lambda_{1},\lambda_{2}\}$.
